# Minimally Invasive Acromioclavicular Joint Stabilization Using an Adjustable Endobutton: A Report of Two Cases

**DOI:** 10.7759/cureus.108554

**Published:** 2026-05-09

**Authors:** Samir Ben Salah, Ayman Ben Abdellah, Achraf Tebbaa El Hassali, Najib Abdeljaouad, Hicham Yacoubi

**Affiliations:** 1 Department of Orthopedic Trauma, Mohammed VI University Hospital, Oujda, MAR; 2 Faculty of Medicine and Pharmacy, Mohammed First University, Oujda, MAR; 3 Department of Orthopedics and Traumatology, Mohammed VI University Hospital, Oujda, MAR

**Keywords:** acromioclavicular joint, dislocation, minimally invasive surgery, shoulder, surgical case reports

## Abstract

Acromioclavicular joint dislocations (ACJD) are frequent shoulder injuries in young, active patients and athletes and can lead to pain, functional impairment, and cosmetic deformity if not adequately treated. Surgical management of high‑grade ACJD remains controversial, with numerous techniques described and no clear gold standard, particularly regarding the balance between stability, invasiveness, and the need for hardware removal.

This study of two cases (Rockwood types III and IV) evaluated a novel minimally invasive technique using an adjustable Endobutton (Smith & Nephew, London, UK) with a single clavicular tunnel via a mini-open deltopectoral approach. At the six‑ and 12‑month follow‑up, both patients showed excellent anatomic reduction maintained over time, no loss of reduction or major complications, and near‑complete functional recovery as assessed by the Oxford Shoulder Score. The technique restores vertical stability, limits surgical morbidity, avoids secondary hardware removal, and adheres to anatomic principles by recreating coracoclavicular ligament function. While these results are encouraging, the small sample size and retrospective design limit the generalizability of the findings, and larger prospective comparative studies are needed to confirm the effectiveness and safety of this approach.

## Introduction

Acromioclavicular joint dislocations (ACJD) account for 9-12% of shoulder injuries, predominantly affecting young, active adults following direct trauma [1-3]. The Rockwood classification guides management: stages I and II (partial ligament sprains) are treated conservatively with good results [2,3].

For stage III injuries, the therapeutic strategy remains controversial, whereas surgery is widely recommended for stages IV-VI, particularly in young, athletic patients [4-6]. Numerous surgical techniques exist (hook plate, Bosworth screw, pins, tendon reconstructions, synthetic ligaments, and cortical button systems such as Endobutton (Smith & Nephew, London, UK) and TightRope (Arthrex, Naples, FL, USA)), used alone or in combination [5,6]. No gold standard has emerged, but recent trends favor anatomic coracoclavicular reconstruction using minimally invasive approaches that allow dynamic stabilization and avoid secondary hardware removal [7-9].

Endobutton/suture-button suspension techniques for acute ACJD (Rockwood types III-VI) generally provide excellent functional outcomes, good radiological stability, and low complication rates and are at least as effective as (if not superior to) hook plates or other rigid fixation devices. Outcomes appear even better when surgery is performed early and when the positioning of the button beneath the coracoid is precise [7,9].

However, the literature remains limited regarding adjustable constructs using a single clavicular tunnel with mini-open passage under the coracoid, allowing progressive tensioning and fine-tuning of reduction. This work reports two cases of high-grade ACJD treated with an adjustable Endobutton using an original, rapid, minimally invasive construct and discusses our results in light of recent data.

## Case presentation

This paper reports our experience with two patients who underwent surgery for ACJD in our orthopedic department using an adjustable Endobutton with an innovative, rapid, and effective construct. Both cases underwent detailed history-taking and complete clinical examination. Radiological assessment consisted of standard shoulder radiographs, including anteroposterior and Zanca views focused on the acromioclavicular joint. Clinical and paraclinical data collection and patient follow-up were ensured using our institutional computerized medical records archiving system.

**Figure 1:**
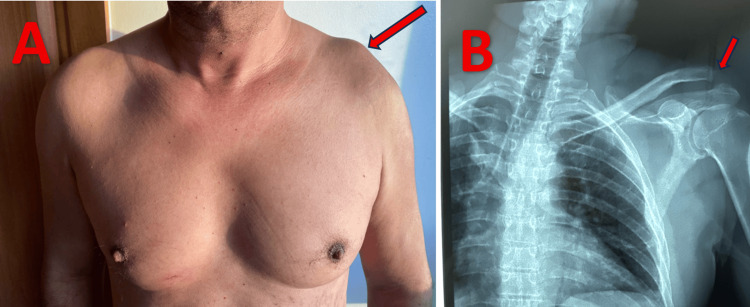
Clinical and imaging features of Case 1 (A) External clinical view demonstrating the dislocation (red arrow). (B) Plain radiograph illustrating a Rockwood type III acromioclavicular joint dislocation (red arrow).

The first case was a 40-year-old male patient with no significant past medical history, who was the victim of a road traffic accident as a motorcyclist hit by a car, with a direct impact mechanism to the left shoulder tip. He presented to the emergency department with pain, functional impairment, and deformity of the left shoulder. Clinical examination revealed tenderness on palpation over the acromioclavicular joint with visible and palpable lateral prominence of the clavicle. A left shoulder radiograph confirmed an ACJD classified as stage IV according to the Rockwood classification (Figure 1).

**Figure 2:**
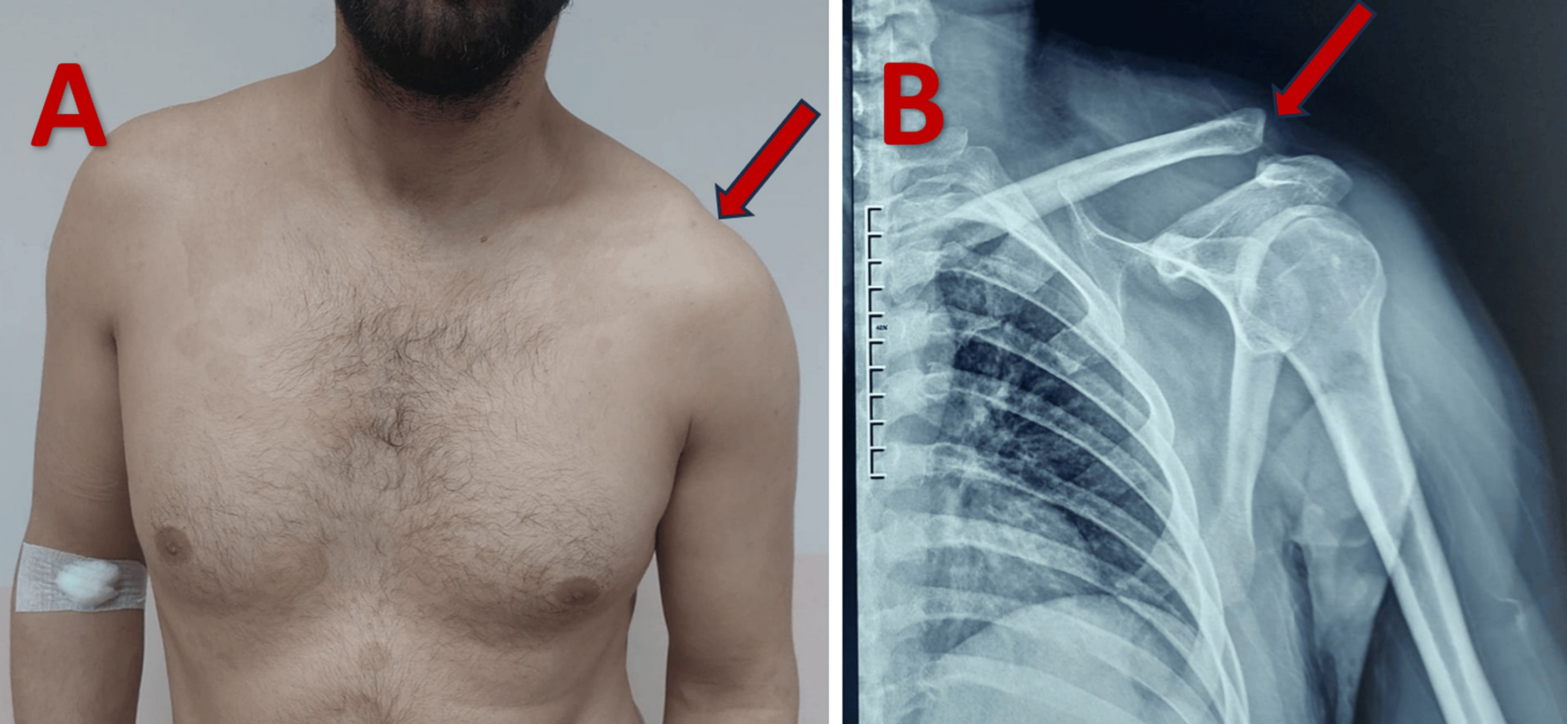
Clinical and imaging features of Case 2 (A) External clinical view demonstrating the dislocation (red arrow). (B) Plain radiograph illustrating a Rockwood type III acromioclavicular joint dislocation (red arrow).

The second case was a 45-year-old male professional driver with no significant past medical history, who was the victim of a road traffic accident as a pedestrian hit by a car and transported to the trauma emergency department by firefighters. Initial examination revealed a painful and shortened left shoulder with impossible passive and active mobility due to pain. Standard shoulder radiographs showed an ACJD classified as stage III according to the Rockwood classification (Figure 2).

**Figure 3:**
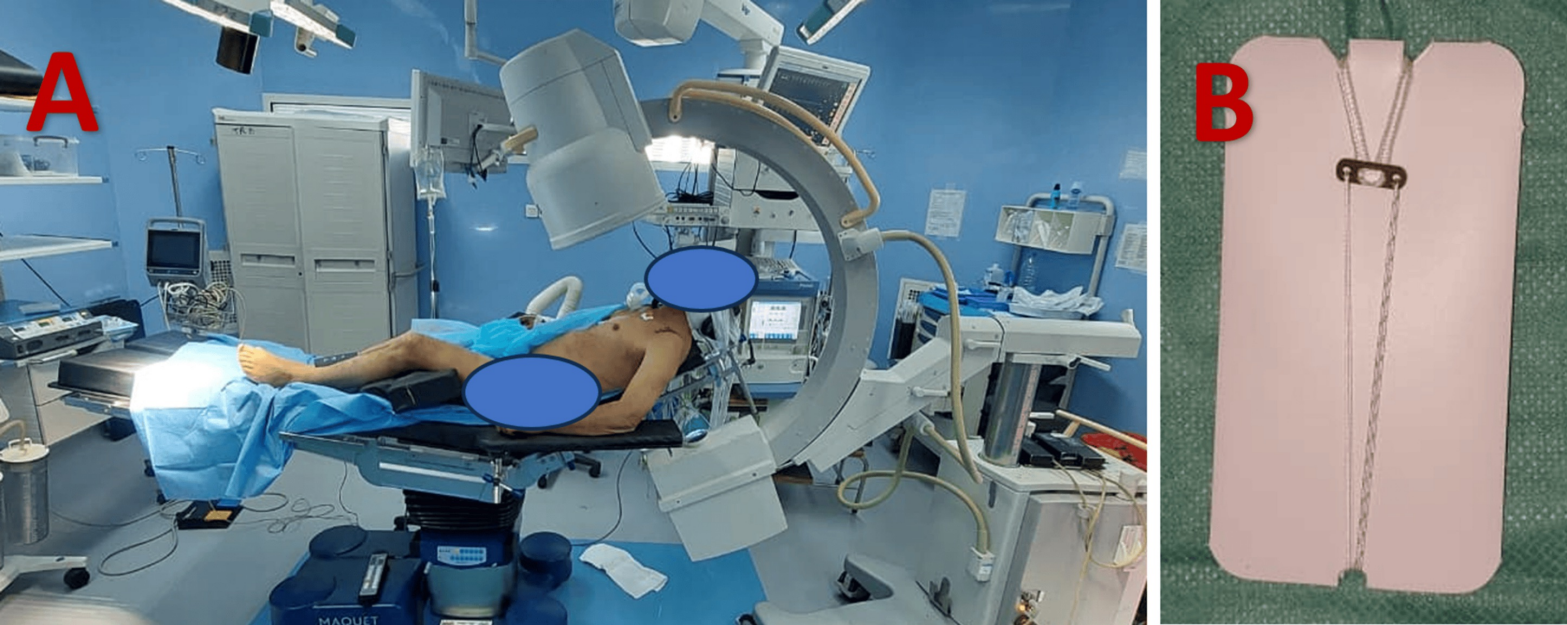
Patient setup and instrumentation (A) Patient positioned in the semi-seated (beach-chair) position. (B) Endobutton devices utilized.

**Figure 4:**
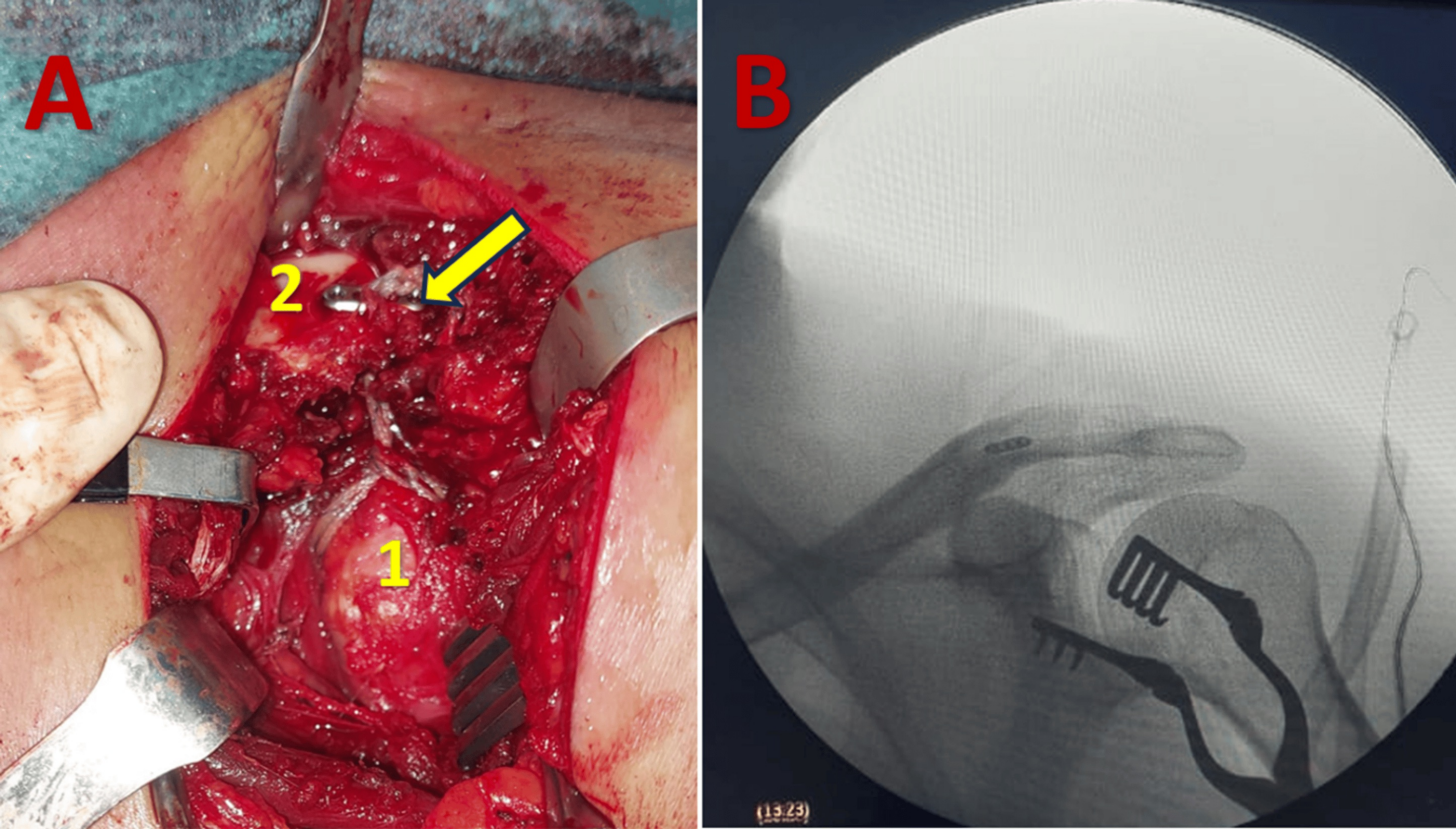
Intraoperative findings (A) Intraoperative view demonstrating the Endobutton construct (1: coracoid process; 2: clavicle; yellow arrow: Endobutton plate). (B) Intraoperative fluoroscopic assessment.

The surgical procedure was performed under general anesthesia with the patients placed in a beach-chair position. The image intensifier was positioned behind the patient, and the surgeon stood facing the patient's shoulder. Using a mini-open deltopectoral approach, after skin incision and hemostasis, the cephalic vein was identified and retracted laterally. Access was gained through the groove between the deltoid medially and the pectoralis major laterally, identifying the coracoid process inferiorly and the acromioclavicular joint superiorly. Subsequently, Endobutton placement proceeded as follows (Figures 3-4). A tunnel was created in the clavicle reamed from superior to inferior with placement of a flat, blunt instrument under the clavicle for neurovascular protection, approximately 3 cm medial to the joint line. A small opening of 3 mm was made with electrocautery under the coracoid process, ensuring the cautery tip was in contact with the inferior cortex of the coracoid process, as soft tissue interposition between the Endobutton loop and the bone can lead to subsequent construct loosening. The Endobutton loop was passed under the coracoid process using a traction suture, followed by passage of the Endobutton plate through the loop to encircle the coracoid process. The plate was then passed through the clavicular tunnel, while the assistant applied pressure with a bone tamp on the lateral part of the clavicle to reduce the dislocation, after which the construct was tightened to maintain reduction. Intraoperative fluoroscopic control was performed to confirm the result.

**Figure 5:**
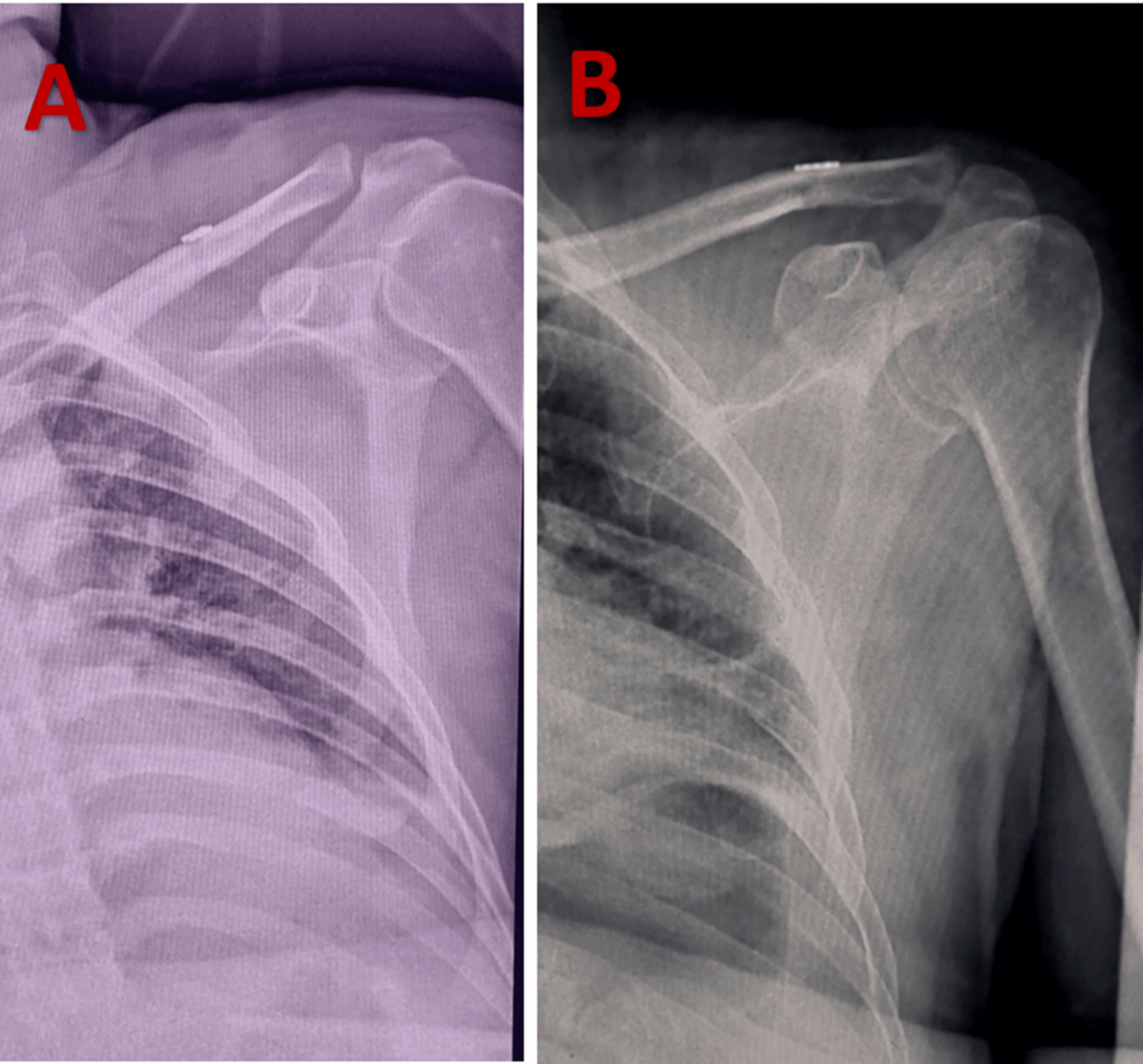
Radiographic follow-up one year after the surgical procedure showing the anatomical reduction of the acromioclavicular joint dislocation (A) Case 1. (B) Case 2.

Postoperative construct control was ensured by standard shoulder radiographs, which demonstrated good ACJD reduction in both cases. With an average follow-up of one year for the first case and six months for the second, we obtained very good functional results based on the Oxford Shoulder Score evaluation (Figure 5).

## Discussion

Our two cases of acute high-grade ACJD (Rockwood types III and IV) in middle-aged men following road traffic accidents are consistent with large Endobutton series [8,9]. Both patients achieved anatomic reduction without radiological loss at follow-up (six months and one year), with excellent Oxford Shoulder Scores aligning with Constant/University of California, Los Angeles (UCLA) scores of 90-100 reported in the literature [10-12].

Recent studies confirm that Endobutton fixation provides excellent vertical stability with Constant scores of 90-96 and low Disabilities of the Arm, Shoulder and Hand (DASH) scores in acute ACJD types III-V [8]. Our results, namely, rapid surgery via a mini-open deltopectoral approach, low morbidity, and rapid functional recovery, align with the trend toward minimally invasive adjustable loop techniques.

Compared to hook plates, which are associated with subacromial impingement, chronic pain, acromial osteolysis, and the need for secondary surgery [13,14], Endobutton/TightRope systems rank among the best compromises for Constant scores, pain relief, and complication rates [6].

However, Endobutton reconstructions have potential complications: early loss of reduction (20-25% in some series), tunnel widening, hardware irritation, and iatrogenic fractures [15,16]. Risk factors include poor tunnel positioning, absence of temporary fixation, and premature loading (<5 weeks) [16]. Our technique limits these risks via a single clavicular tunnel 3 cm from the joint line, controlled coracoid opening, manual reduction during tightening, and systematic fluoroscopic control. The absence of reduction loss in our cases likely relates to precise tunnel trajectory and correct button positioning [5,6]. No fractures or hardware irritation occurred, consistent with low-profile series [8].

Prompt management (within 2-3 weeks) yields better outcomes [1,8]; both our cases were operated in the acute phase. Our construct resembles percutaneous/mini-open techniques combining a single tunnel, loop passage under the coracoid, and adjustable Endobutton tightening [5,8], offering limited exposure, soft tissue preservation, no secondary hardware removal, and intraoperative tension fine-tuning, advantages aligned with adjustable loop systems [8,17].

Limitations include only two patients, no control group, and no long-term follow-up. Larger comparative series are needed to confirm benefits over other techniques (autograft, Ligament Augmentation and Reconstruction System (LARS), LockDown, etc.) [18,19], as well as the evaluation of long-term reduction, osteoarthritis, and functional satisfaction beyond 2-3 years. Despite these limitations, our results support minimally invasive cortical button techniques as safe, effective, and potentially superior for acute high-grade ACJD [1,8-10].

## Conclusions

The experience reported in this work, although involving a limited number of patients, is consistent with this data: the minimally invasive technique using an adjustable Endobutton offers stable reduction, near-complete functional recovery, and no major complications in the short term. The main points of vigilance remain precise coracoid button positioning, prevention of osteolysis, and adherence to a progressive rehabilitation protocol, identified as key factors to avoid loss of reduction.

Nevertheless, prospective comparative studies on larger series with extended follow-up are necessary to confirm the durability of these results and to precisely define the role of this technique within the therapeutic arsenal for ACJD.
